# Supplementary material for: Conventional and Unconventional Therapeutic Strategies for Sialidosis Type I
Source: J Clin Med. 2020 Mar 4;9(3):695. doi: 10.3390/jcm9030695 (PMC7141319; doi:10.3390/jcm9030695)
Supplement: Supplementary file 1 [file jcm-09-00695-s001.pdf]

Supplementary Material

Conventional and Unconventional Therapeutic Strategies for Sialidosis Type I

Rosario Mosca [1,8], Diantha van de Vlekkert [1,8], Yvan Campos [1], Leigh E. Fremuth [1,2], Jaclyn Cadaoas [3], Vish Koppaka [3], Emil Kakkis [3], Cynthia Tifft [4], Camilo Toro [5], Simona Allievi [6], Cinzia Gellera [6], Laura Canafoglia [6], Gepke Visser [7], Ida Annunziata [1] and Alessandra d'Azzo [1,*]

1 Department of Genetics, St. Jude Children's Research Hospital, Memphis, TN, 38105, USA.
2 Department of Anatomy and Neurobiology, College of Graduate Health Sciences, University of Tennessee Health Science Center, Memphis, TN, 38163, USA
3 Ultragenyx Pharmaceutical, Novato, CA, 94949 USA
4 Office of the Clinical Director & Medical Genetics Branch, National Human Genome Research Institute, National Institutes of Health (NHGRI), Bethesda, MD, 20892, USA
5 Undiagnosed Disease Network, National Human Genome Research Institute, National Institutes of Health, Bethesda, MD, 20892, USA.
6 Neurophysiopathology, Fondazione IRCCS Istituto Neurologico Carlo Besta, Milan, 20133, Italy
7 Department of Metabolic Diseases, Wilhelmina Children's Hospital, University Medical Center Utrecht, Utrecht, 3584 CX, The Netherlands.
8 Equal contributors
* Correspondence: sandra.dazzo@stjude.org; Tel.: +xx-xxxx-xxx-xxxx

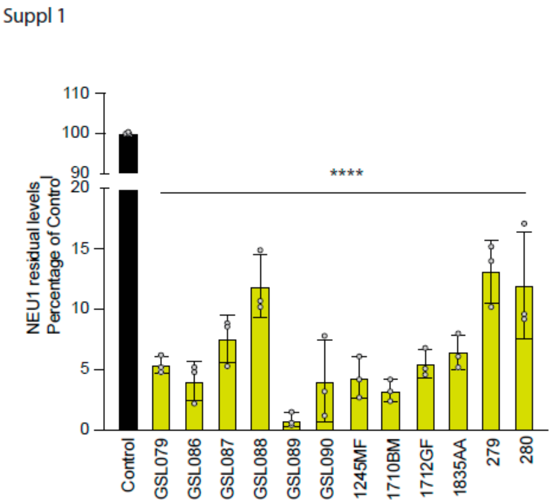

Supplementary Figure S1. Quantification of endogenous NEU1 levels in the 12 type I sialidosis fibroblasts, normalized to the loading control ($n = 3$). Graphs are presented as mean ± SD. $^{****}p < 0.0001$.

Suppl 2

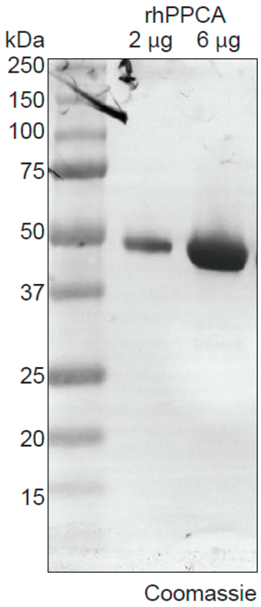

Supplementary Figure S2. Coomassie-stained membrane loaded with recombinant human PPCA (rhPPCA).

Supple 3

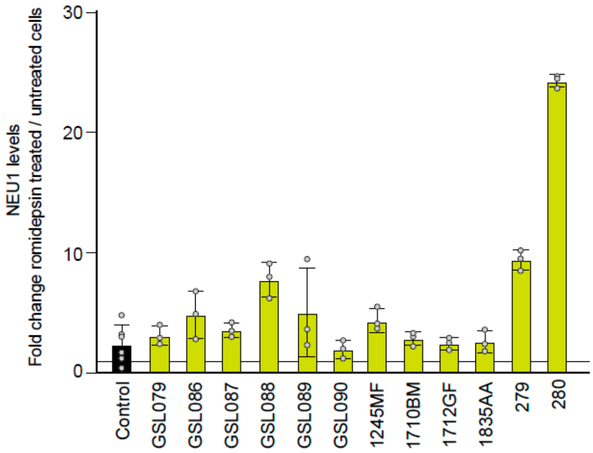

Supplementary Figure S3. Quantification of NEU1 levels in the 12 type I sialidosis fibroblasts after treatment with romidepsin calculated as fold of increase between treated versus untreated cells and normalized to loading control ($n = 3$). Graphs are presented as mean ± SD.

Supple 4

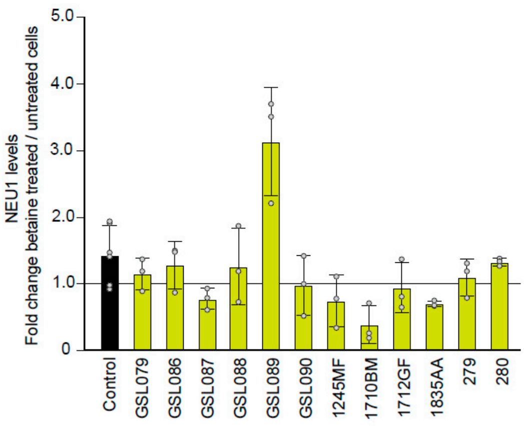

Supplementary Figure S4. Quantification of NEU1 levels in the 12 type I sialidosis fibroblasts after treatment with betaine calculated as fold of increase between treated versus untreated cells and normalized to loading control ($n = 3$). Graphs are presented as mean ± SD.

Supple 5

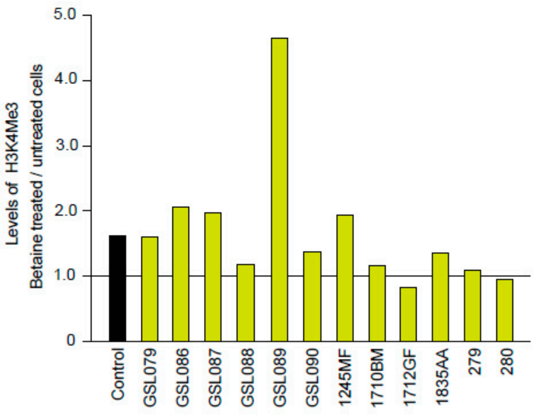

Supplementary Figure S5. Quantification of H3K4me3 levels in the 12 type I sialidosis fibroblasts after treatment with betaine calculated as fold of increase between treated versus untreated cells and normalized to loading control. Graphs are presented as mean ± SD.

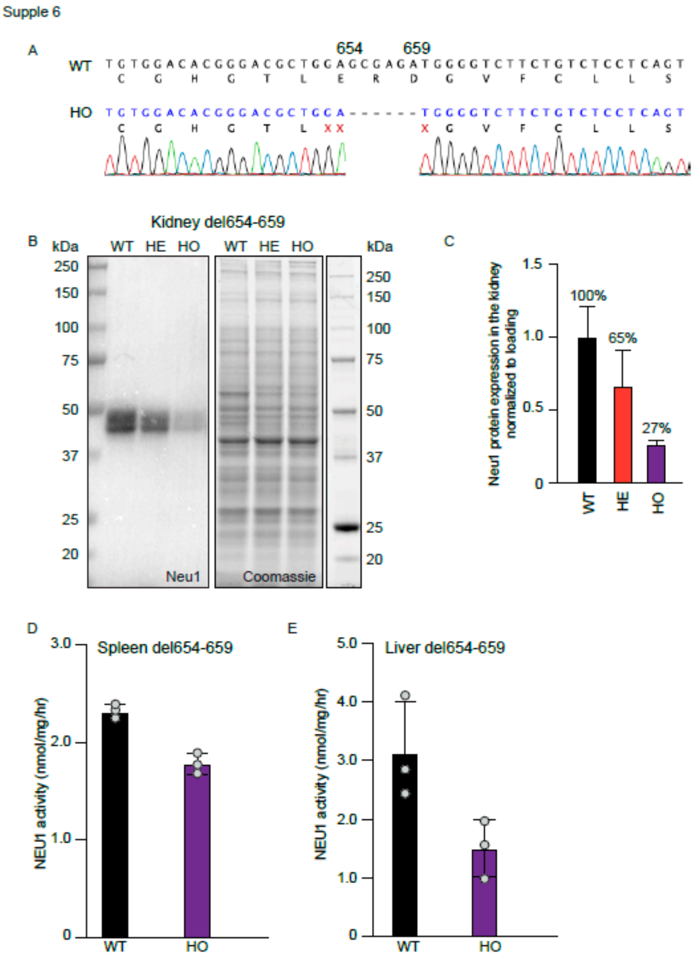

Supplementary Figure S6. Residual Neu1 activity in the *Neu1*$^{del654\text{-}659}$ (HO) model. (**A**) Electropherogram showing the 6 base pairs deletion. (**B**) Neu1 activity and (**C**) Quantification of Neu1 protein levels tested in kidneys of mice homozygous for the del654-659 mutation. Neu1 activity in (**D**) spleen and (**E**) liver of the same mouse model. Graphs are presented as mean ± SD.
